# Elementary calcium release events in the skeletal muscle cells of the honey bee *Apis mellifera*

**DOI:** 10.1038/s41598-021-96028-w

**Published:** 2021-08-18

**Authors:** Claude Collet, Mercédès Charreton, Laszlo Szabo, Marianna Takacs, Laszlo Csernoch, Peter Szentesi

**Affiliations:** 1 UR406 Bees and Environment, French National Institute For Agriculture, Food and Environment, INRAE, 84914 Avignon, France; 2 Department of Electrical Engineering, Sapientia Hungarian University of Transylvania, Tîrgu Mureș, Romania; 3 Department of Animal Science, Faculty of Agricultural and Food Sciences and Environmental Management, University of Debrecen, Debrecen, Hungary; 4 Department of Physiology, Medical Faculty, University of Debrecen, Debrecen, Hungary

**Keywords:** Physiology, Biophysics, Permeation and transport

## Abstract

Calcium sparks are involved in major physiological and pathological processes in vertebrate muscles but have never been characterized in invertebrates. Here, dynamic confocal imaging on intact skeletal muscle cells isolated enzymatically from the adult honey bee legs allowed the first spatio-temporal characterization of subcellular calcium release events (CREs) in an insect species. The frequency of CREs, measured in x–y time lapse series, was higher than frequencies usually described in vertebrates. Honey bee CREs had a larger spatial spread at half maximum than their vertebrate counterparts and a slightly ellipsoidal shape, two characteristics that may be related to ultrastructural features specific to invertebrate cells. In line-scan experiments, the histogram of CREs’ duration followed a bimodal distribution, supporting the existence of both sparks and embers. Unlike in vertebrates, embers and sparks had similar amplitudes, a difference that could be related to genomic differences and/or excitation–contraction coupling specificities in honey bee skeletal muscle fibres. The first characterization of CREs from an arthropod which shows strong genomic, ultrastructural and physiological differences with vertebrates may help in improving the research field of sparkology and more generally the knowledge in invertebrates cell Ca^2+^ homeostasis, eventually leading to a better understanding of their roles and regulations in muscles but also the myotoxicity of new insecticides targeting ryanodine receptors.

## Introduction

Bees are very efficient pollinator insects, and are thus crucial both for plant biodiversity maintenance and agricultural food production. Amongst bees, owing to their easy domestication, honey bees have long been used as a research model in various fields, ranging from cell physiology to inter-individual communication and sociability^1,2^. Moreover, the high levels of mortality in honey bee’s populations observed worldwide in the last few years prompted researchers to further study this model with the hope of understanding origins of this decline. Honeybee cells provide valuable models to study calcium signalling and intracellular calcium homeostasis in insects^3–11^. Calcium is an essential intracellular second messenger which plays pivotal roles in neuromuscular cellular processes such as signal transduction and modulation in motor or sensory neurons, contraction in cardiac, smooth, and skeletal muscle cells. More generally, Ca^2+^ plays a major role in development/maturation, cell shape modulation and migration, cell division, cell-membrane repair, hormonal secretion, and gene expression in virtually all cell types. In the honey bee, macroscopic intracellular Ca^2+^ variations have been recorded with Ca^2+^-imaging techniques in neurons from central brain structures such as mushroom bodies^3,4^ or antennal lobes^7,8^. Recently, Ca^2+^ was also monitored in peripheral sensory neurons from the antennae, i.e. the ‘nose’ of honeybees^10^. Global Ca^2+^ fluctuations were also studied in photoreceptor cells from the honey bee compound eye^11^. Finally, cytoplasmic Ca^2+^ transients (i.e. transient Ca^2+^ increases that can be elicited by a stimulation) and excitation–contraction coupling have been described in bee skeletal muscle cells as well, using fluorescent dyes and electrophysiological techniques^5,6^. Recently, studying intracellular Ca^2+^ signalling in bees and pollinators gained renewed interest because new classes of insecticides targeting several molecular players involved in Ca^2+^ homeostasis have been launched on the pesticides market and are now widely used in agriculture. Notably, most recent diamide type insecticides such as flubendiamide and chlorantraniliprole induce detrimental effects in honey bees by anarchically mobilizing Ca^2+^ from intracellular stores through Ca^2+^ release channels in neurons from the antenna and in skeletal muscle cells from the leg^9,10^.

A major molecular player in Ca^2+^ signalling is undoubtedly the ryanodine receptor. The molecular identity of this receptor was unravelled thanks to the botanical alkaloid ryanodine, present in root extracts of the plant species *Ryana*, which was used in the 50’s as a natural insecticide (now withdrawn owing its’s toxicity towards environment). Ryanodine receptors are huge macromolecular complexes functioning as ion channels to provide a route for Ca^2+^ to be massively transferred on demand from intracellular storage organelles (sarco and endoplasmic reticulums) to the cytoplasmic compartment^12,13^. Increasing the intracellular cytoplasmic Ca^2+^ concentration ([Ca^2+^]_i_) from submicromolar to tenths of micromolar ranges thus allows Ca^2+^ to play its multiple regulatory roles^14,15^. At rest, [Ca^2+^]_i_ is tightly regulated and maintained at around 100 nM to avoid the toxicity of sustained Ca^2+^ increases and this low resting [Ca^2+^]_i_ provides both a wide dynamic range and a high signal-to-noise ratio. Optical and electrophysiological techniques evolved and allowed characterizing cytoplasmic Ca^2+^ fluctuations with continuously increased acuity along with recent years. Nowadays, laser-scanning confocal microscopes allow detecting optically subcellular cytoplasmic Ca^2+^ fluctuations in the µm spatial and in the ms temporal ranges with the help of fast and high-affinity Ca^2+^ dyes (Ca^2+^ indicators). These localized fluctuations are believed to originate from the release of Ca^2+^ by one or a cluster of several ryanodine receptors and in striated muscles, they are considered as the unitary bricks of voltage-induced global Ca^2+^ release^16^. Localized elevation in [Ca^2+^]_i_ can also fuse to initiate propagating macroscopic Ca^2+^ waves and have specific cytosolic localizations such as the perinuclear space where they can modulate nuclear Ca^2+^ independently of the bulk cytosolic Ca^2+^^17^. So far, local fluctuations in [Ca^2+^]_i_ have been studied only in muscle cells from vertebrate species. First discovered in cardiac myocytes^18^, and originally termed Ca^2+^ sparks, similar signalling events were identified later in smooth muscle cells, skeletal muscle fibres, neurons and oocytes. The role and regulation of mechanisms of these spatially localized fluctuations are however elusive and remain to be fully identified with a rather unique research field dedicated to their study: ‘sparkology’^16,17^. This research field is essential as these signals are frequently altered in genetic diseases linked to mutations in the ryanodine receptor genes (ryanodinopathies). Even under physiological conditions, it soon turned out that localized Ca^2+^ fluctuations had many temporal patterns, not only an archetypal steep increase (~ 5 ms) and a fast mono-exponential decrease, and new terms emerged to better describe this signals diversity e.g. embers, macrosparks, compound sparks, bursts, sparklets…^19^. A more general term is now used to depict these [Ca^2+^]_i_ fluctuations : elementary Ca^2+^ release events (CREs).

While CREs have been identified in many tissues from several vertebrate species (rat, mouse, frog and fish), to our knowledge, they have never been identified in an invertebrate tissue. Here, using honey bee as a model, we describe for the first time elementary CREs in an insect species and compare their spatiotemporal characteristics in the light of interspecific physiological and ultrastructural cellular differences. Part of this work was presented to the Biophysical Society^20^.

## Material and methods

### Isolation of leg muscle cells

Muscle fibres were prepared from young adult domestic honey bees (*Apis mellifera*), as described earlier^5^, with slight modifications. Newborn bees were collected outdoor, directly on a frame of a hive and used during the following 4 days. Bees were chilled (~ 4 °C) until lethargic immobility and then killed by decapitation. Tibias from the third pair of legs were dissected in a Tyrode’s solution without calcium (in mM: NaCl 140, KCl 5, MgCl_2_ 2, HEPES 10, pH 7.2), cut longitudinally and pinned open. Tibias were bathed 20 min in a dissociation solution at 37 °C (Tyrode’s solution without calcium containing four enzymes: collagenase type 1, trypsin, papain, and pronase at concentrations 0.5, 1.5, 1.5 and 1 mg/ml, respectively). After rinsed in Tyrode’s solution without calcium and normal Tyrode (2 mM CaCl_2_), muscle masses were triturated with a pipette and dispersed in the central well of a clean glass bottom dish (MatTek Corp., Ashland, MA, USA). Cell were loaded for 30 min with the cell permeant dye Fluo-8-AM (5 µM).

### Measurement of Ca^2+^ release events (CREs) in intact bee muscle fibres

**Figure 1:**
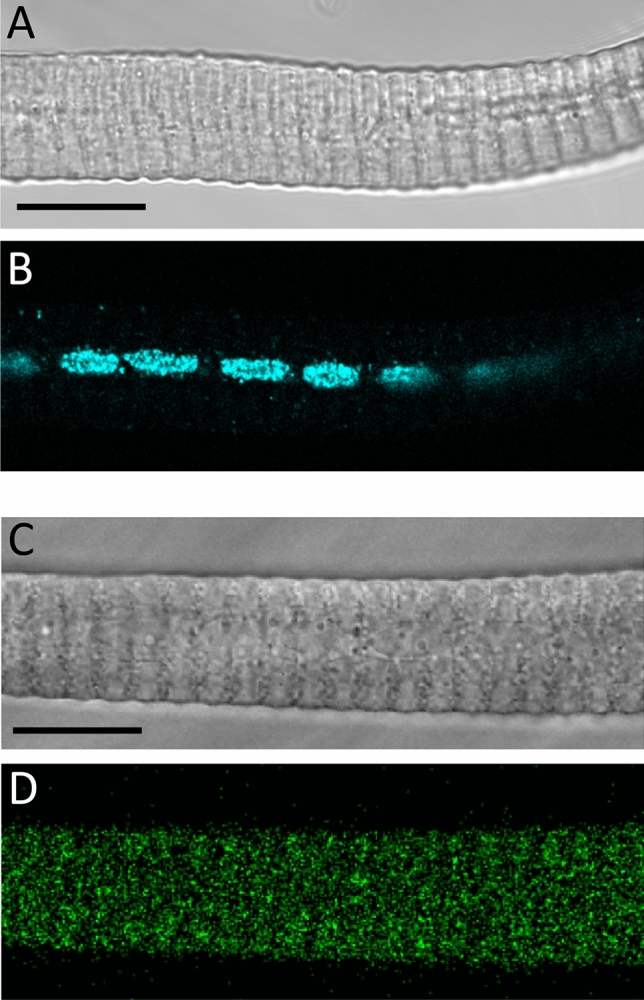
General structure of bee muscle fibres. (**A)** Transmitted light for a honey bee tibia muscle fibre (mean sarcomere length 4,9 µm). (**B**) Confocal longitudinal section through the fluorescent central row of nuclei labelled with Hoechst stain (same fibre as in A). (**C**) Transmitted light for a Fluo-8 stained fibre (mean sarcomere length 4,4 µm). (**D**) Confocal fluorescence image of the fibre shown in C. Scale bars = 20 µm.

Fluo-8-AM loaded cells were kept in normal Tyrode’s solution and observed with a laser scanning confocal microscope Zeiss LSM 5 LIVE (Zeiss, Oberkochen, Germany) equipped with a Plan-Neofluar 40 × oil immersion objective (NA = 1.3). Fluo-8 was excited with the 488 nm wavelength of an argon laser and the emitted fluorescent light was measured through a band-pass filter (505–570 nm) and digitized at 12 bit. First the fibres were examined by eye and the fibres showing spontaneous activity were recorded with series of 512 × 512 (*XYT*) images, captured at 1 Hz. The pixel size was 0.328 µm and the pixel dwell time was 1 ms. CRE detection and analysis were performed using methods and algorithms previously described^21,22^. In brief, the region of the fibre and the background was defined on the first image of each series. The averaged background fluorescence was subtracted from each pixel of all images in one series. Ca^2+^ release events were detected by the stationary wavelet method. The filtering was made by soft thresholding wavelet detection. Finally the amplitude and full width at half maximum (FWHM) of the sparks were calculated. Two FWHM values were determined: perpendicular to (X) and parallel (Y) with the Z-lines. To detect the Z-lines the frequency spectrum in each line of all images was calculated using fast Fourier transform. To remove the Z-lines from the images, inverse FFT of the frequency components corresponding to sarcomeres was used. Bleaching correction was performed on all images by calculating the change in fluorescence intensity of each pixel in time. Finally images were normalized using stationary wavelet transform. The event frequency was determined by counting the number of events on all images from a series. Events that appeared on consecutive images at the same location were counted as a single event. To get the frequency the number of events was normalized to fibre area and duration of recording. To assess the amount of Ca^2+^ released during a CRE, the Signal Mass (SM) was calculated as described earlier^23^. To estimate FWHM in the z direction (i.e. the optical axis) in two-dimensional measurements, the average of the two FWHMs in the focal plane (x and y directions) was used. Figure 1 and Supplementary Video 1 were made under the ImageJ software (v1.52a, Rasband, W.S. ImageJ, U.S. National Institutes of Health, Bethesda, Maryland, USA, https/imagej.nih.gov/ij/, 1997–2018).

Line-scan images (*XT*) were taken at 1 or 2 ms/line and the pixel size was 0.328 µm. In some cases 0.2 ms/line was used to study the temporal properties of CREs in high time resolution. The length of the line was variable and depended on the actual fibre width. The images (F[x,t]) were normalized to baseline fluorescence (F_0_[x]). Line-scan images containing spontaneous calcium release events were analysed using an automatic event detection program^21^, which calculated the amplitude (ΔF/F_0_), rise time, full width at half maximum (FWHM), and full duration at half maximum (FDHM) of the identified events. An event was identified as an ember if it had a clear plateau and was longer than 40 ms in time. Otherwise, it was considered as a spark. Events with an amplitude smaller than 0.1 ΔF/F_0_ units were excluded from the analysis. All measurements were carried out at room temperature (22–23 °C).

### Depolarization-evoked Ca^2+^ transients measurements

Ca^2+^ transients were elicited on CRE free (silent) intact fibres with field stimulation and recorded in line-scan mode. Two platinum electrodes were placed adjacent to the fibres and 50 ms long depolarizations with amplitude ranging between 40 and 80 V were used to evoke Ca^2+^ transients (S88 Stimulator, Grass Technologies, Warwick, RI, USA). Whole-cell Ca^2+^ transients were recorded in patch-clamp experiment as described earlier^5^.

### Nuclei staining

Fibres were stained with Hoechst 33258 (5 μg/ml) in Tyrode during 30 min. After being loaded, fibres were rinsed twice in Tyrode’s solution and nuclei were visualized under a Zeiss LSM710 confocal inverted microscope with a 63 × 1.42 NA objective (Zeiss, Oberkochen, Germany).

### Resting membrane potential measurement

Single muscle fibres were superfused with normal Tyrode’s at room temperature (24 °C). Membrane potentials were recorded using 3 M KCl filled sharp glass microelectrodes with tip resistances ranging between 30 and 40 MΩ only in cells with intact surface membrane and clear cross striations. The electrodes were connected to the input of a Multiclamp-700A amplifier (Axon Instruments, Foster City, CA, USA) under current-clamp conditions. Membrane potentials were digitized at 100 kHz using Digidata 1322 A/D card (Axon Instruments) under software control (pClamp 9.2, Axon Instruments, Foster City, CA, USA) and stored for later analysis.

### Statistical analysis

Pooled data were expressed as mean ± standard error (SE) of the mean. The differences were assessed using one-way analysis of variance (ANOVA) and all pairwise multiple comparison procedures (Student–Newman–Keuls method). A p-value of less than 0.05 was considered statistically significant. Graphpad Prism 6.0 software (GraphPad software, San Diego, USA) was used for statistical analysis and non-linear regression.

## Results

Honey bee intact skeletal muscle fibres isolated from the legs present a nice sarcomere striation under light microscope and the nuclei are located in the centre of the fibres^5^ instead of right underneath the sarcolemma as in other adult organisms like amphibians and mammals (Fig. 1A, B, Hoechst staining). The axial row of nuclei give them a so-called ‘tubular’ appearance in histological transverse sections. The sarcomere pattern in healthy muscle fibres used in this study showed a period of ~ 4 µm, indicating a relaxed status for the contractile machinery. Their membrane potential, measured with sharp microelectrodes (-58.0 ± 6.9 mV, n = 3) was consistent with measurements made in other insect species^24^ and in enzymatically-isolated mammalian muscle fibres that are polarized around -60 mV^25–27^. An extensive t-tubular organization has been demonstrated earlier in these non-flight muscle fibres, as well as functional Ca^2+^ and K^+^ ionic channels and excitation–contraction coupling^5,28^. Here, examining Fluo-8-loaded leg fibres in confocal fluorescence (Fig. 1C, D) revealed that around one fifth of fibres produced spontaneous calcium release events when bathed in a physiological extracellular solution (Tyrode’s solution).

**Figure 2:**
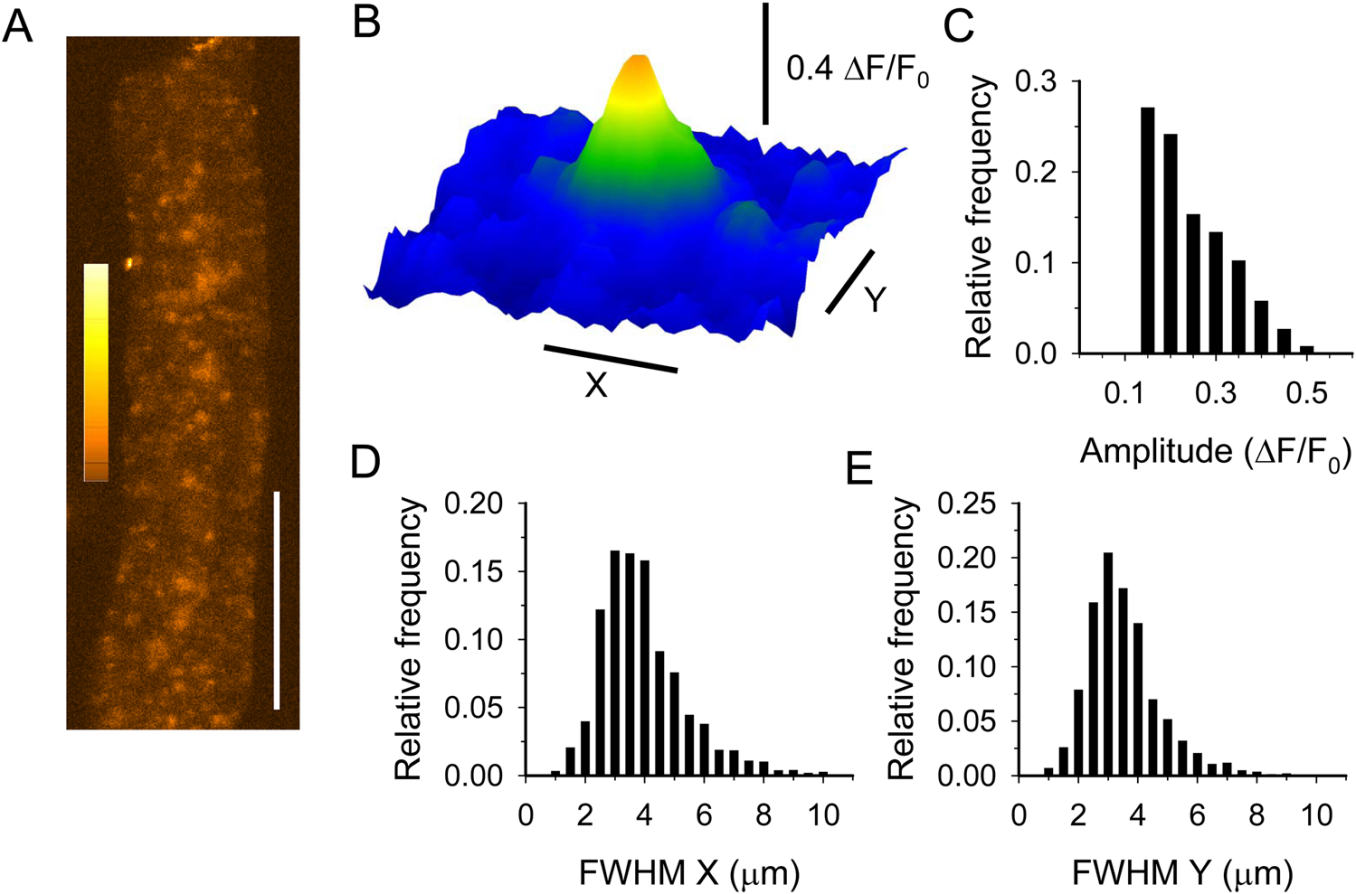
Spontaneous elementary calcium release events in two dimensional (XY) experiments. (**A**) Sum of 30 background corrected images of a Fluo-8 loaded fibre representing 30 s. (**B)** A representative spark. Histograms of parameters of calcium sparks: amplitude (**C**), FWHM perpendicular (D) and parallel (E) to the fibre axis. Calibration on panel A is 50 µm. Calibrations on panel B are 5 µm.

In spontaneously active fibres, localized calcium release events appeared randomly in time and space (Supplementary video 1, Fig. 2A). The events appearing in *XYT* image sequences showed classical spark shape (Fig. 2B). The analysis of a number (4898) of CREs revealed that their average amplitude is quite small (0.220 ± 0.001 ΔF/F_0_), but some higher amplitude events (up to 0.6 ΔF/F_0_) were observed as well (Fig. 2C). This amplitude can be compared to ~ 1–2 ΔF/F_0_ global Ca^2+^ transients we observed during field stimulations (Supplementary Fig. S1) and previously measured under voltage-clamp in bee muscle cells loaded with Fluo-3^5^. The average frequency of CREs was high (1696 ± 367 Hz/mm^2^, n = 25 fibres). The CREs were wide in space since both FWHMs (X and Y) were greater than 3 µm in average (3.73 ± 0.02, and 3.30 ± 0.02 µm, respectively). The probability histograms of FWHMs (Fig. 2D and E) show that some events had a broad size (> 10 µm). The shape of honey bee CREs in space were not completely circular. The average ratio of FWHMs (X/Y) were significantly greater than 1 (1.22 ± 0.01; p < 0.001). The signal mass (SM), calculated to estimate the amount of Ca^2+^ released during a CRE, was 14.52 ± 0.29 µm^3^ on average.

**Figure 3:**
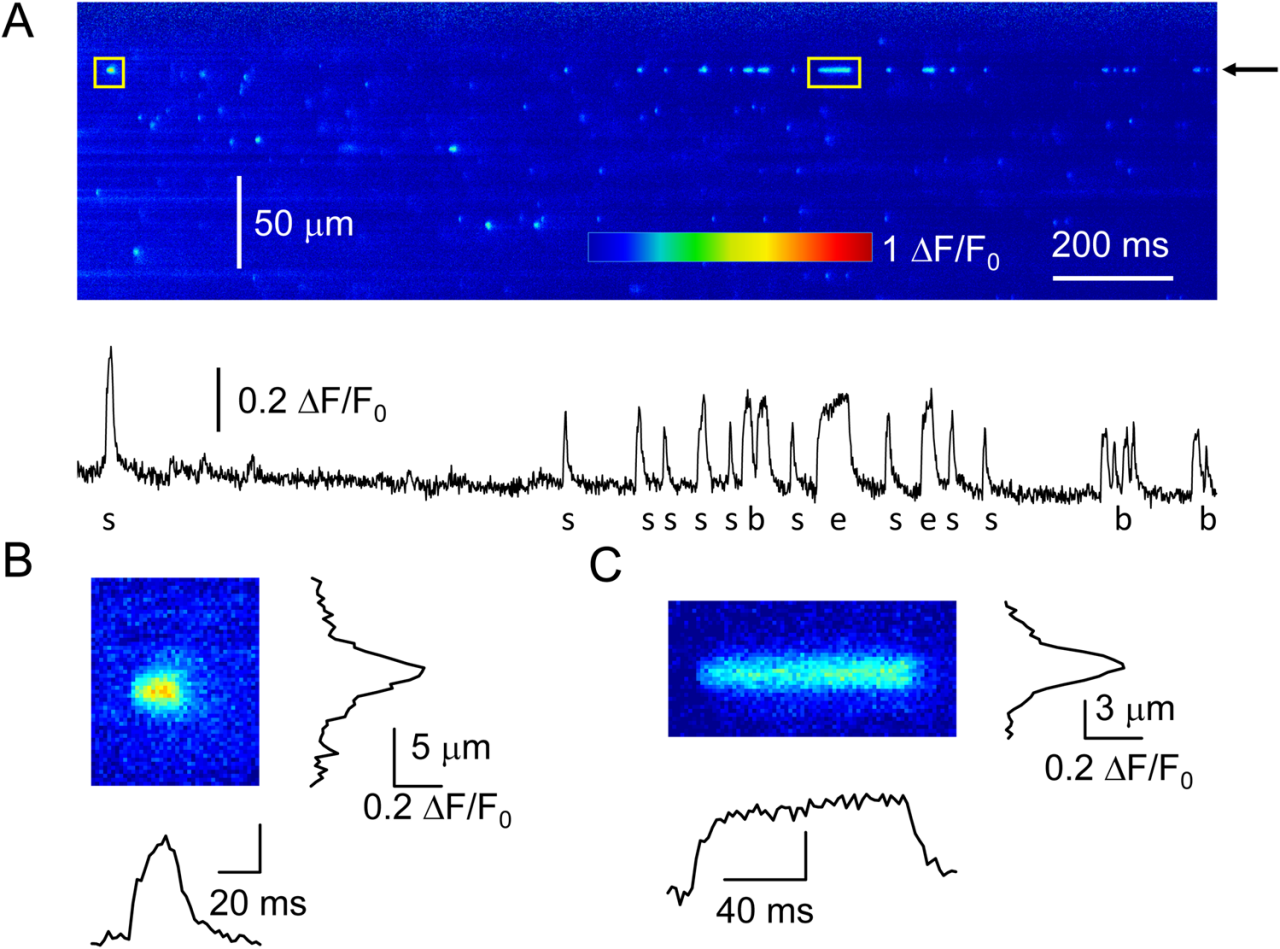
Sparks and embers on line-scan image. (**A**) Representative background corrected line-scan image measured with 2 ms/line scanning speed. The trace below the image was calculated by averaging 5 lines at the position marked by the arrow. Enlarged image of a spark (**B**) and an ember (**C**) with its spatial and time profile. The positions of the events are marked with yellow rectangular in panel A.

To study elementary CREs in more details, we performed line-scan experiments. Figure 3A shows a representative background-corrected line-scan image in pseudo colour. This fibre was very active, and several sparks (Fig. 3B) and long lasting embers (Fig. 3C) were observed in this record. Most CREs appeared randomly in space and time. However, there were some frequently firing sites which generated more than one event in the same place. The trace calculated from Fig. 3A at the position marked by an arrow, shows a representative example of this phenomenon. The time profile of the active calcium release unit demonstrates that the spontaneous event originating from the same place can be a spark, an ember, or a burst^29^.

**Figure 4:**
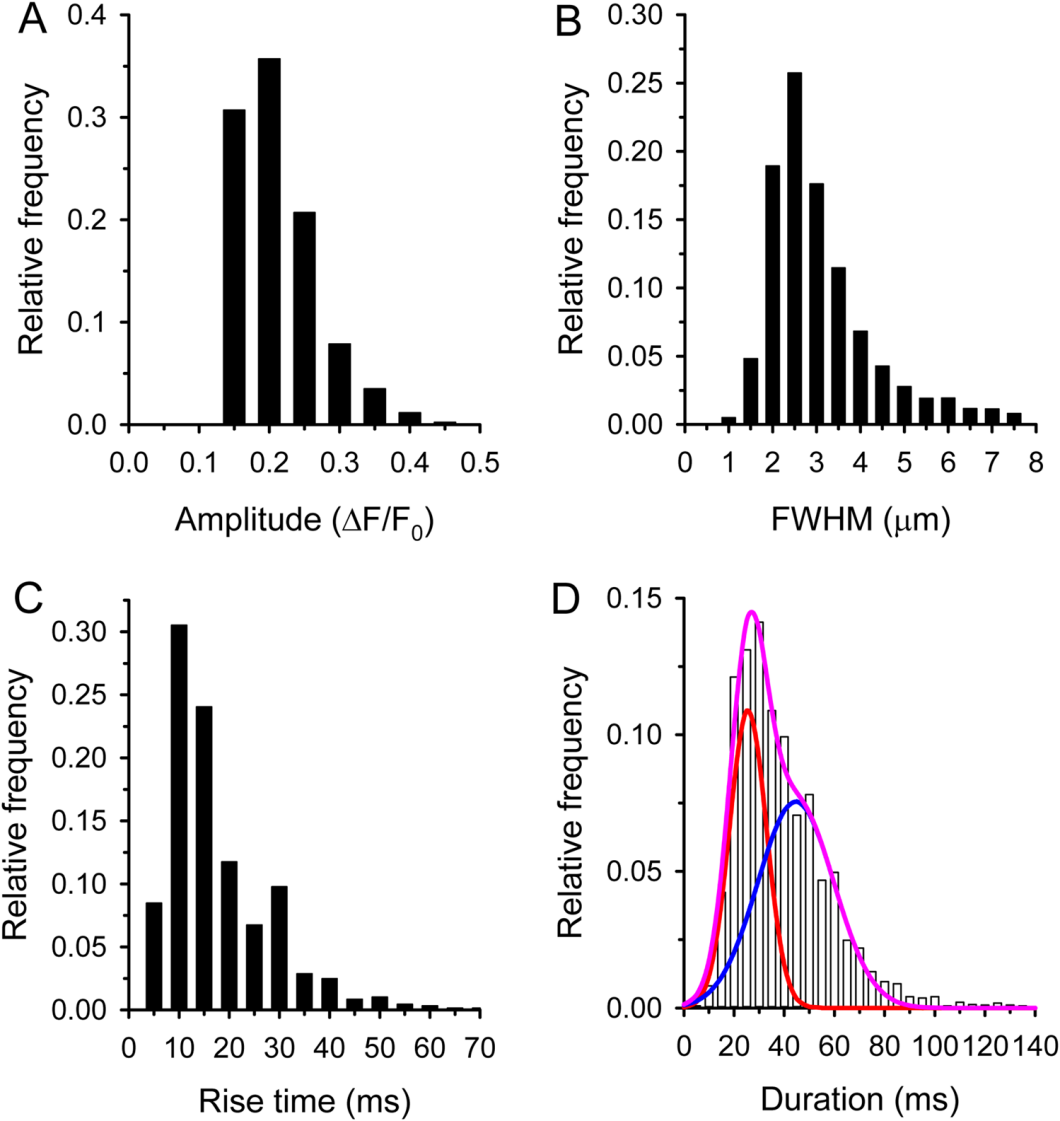
Parameters of calcium release events. Histogram of amplitudes (**A**), FWHMs (**B**), rise times (**C**), and durations (**D**). The number of calcium release events was 3338. The parameters of Gaussian fits in panel D are 0.109 and 0.076 for the amplitude and 25.54 and 44.26 s for the mean to spark (red) and embers (blue), respectively. Pink line represents the sum of the two (red and blue) Gaussian functions.

First, the spatiotemporal parameters of CREs were calculated supposing a homogeneous population (Table 1, Fig. 4). However, as it is clearly visible in Fig. 3 there are some long lasting events, called traditionally embers^30^. The histogram of duration of all CREs was indeed well fitted by the sum of two Gaussian functions (Fig. 4D). The peak position of the first Gaussian was found at 25.5 ± 0.5 ms, while the second function peaked at 44.3 ± 3.9 ms. The intersection of the two Gaussian functions was at around 40 ms and this value was thus chosen as the threshold of duration to distinguish a spark from an ember (see Table 4, column entitled “Threshold duration of embers”). All CREs with a duration longer than 40 ms were categorized as embers.

**Table 1:** Characteristics of honey bee elementary calcium release events detected in line-scan (*XT*) experiments.

(22 fibres)	Amplitude (ΔF/F_0_)	Rise time (ms)	FWHM (µm)	FDHM (ms)	Signal mass (µm^3^)	Frequency (mm^-1^ s^-1^)
All events (3338)	0.185 ± 0.001	15.88 ± 0.20	2.82 ± 0.02	35.82 ± 0.34	7.41 ± 0.19	265.1 ± 15.0
Sparks (2244)	0.185 ± 0.001	10.45 ± 0.11	2.65 ± 0.02	25.52 ± 0.16	6.00 ± 0.20	174.0 ± 14.4
Embers (1094)	0.186 ± 0.002	27.02 ± 0.41***	3.18 ± 0.04***	56.95 ± 0.58***	10.29 ± 0.41***	63.1 ± 6.6***

Events with less than 0.1 amplitude were discarded. All events with greater than 40 ms duration were classified as ember. The numbers in parenthesis are the number of event analysed.

***Denotes significant differences from spark, p < 0.001.

FWHM – full width at half maximum, FDHM – full duration at half maximum.

**Figure 5:**
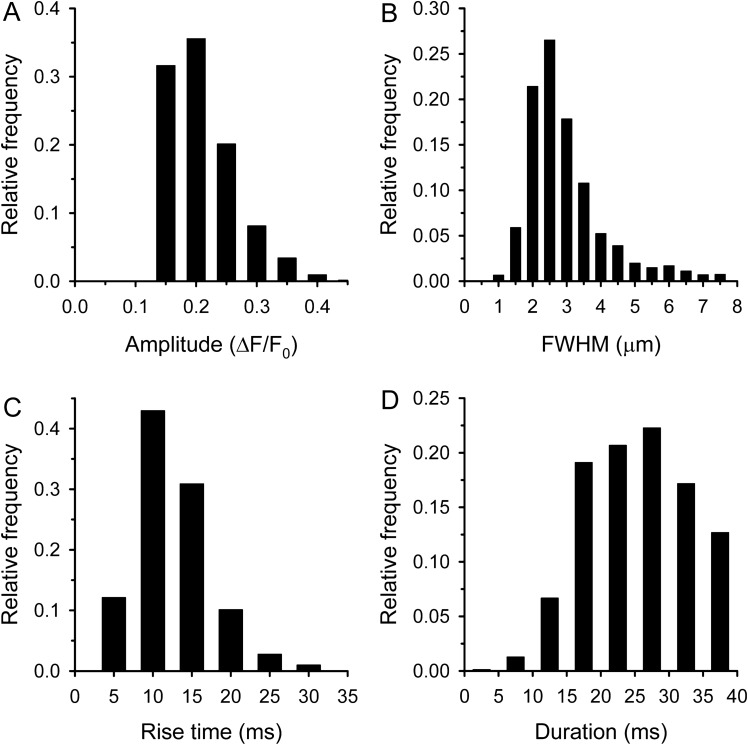
Parameters of calcium sparks. Histogram of amplitudes (**A**), FWHMs (**B**), rise times (**C**), and durations (**D**). The number of sparks was 2244.

Based on this classification we found that 67% of the events were sparks, with a high frequency (Table 1). The average amplitude of sparks was 0.185 ± 0.001 ΔF/F_0_, but the histogram of amplitudes shows some events with higher amplitudes up to 0.4 ΔF/F_0_ (Fig. 5A). The average FWHM of sparks was found to be 2.65 ± 0.02 µm, interestingly we found some very wide sparks with a FWHM greater than 7 µm (Fig. 5B). The distribution of rise times clearly shows that the majority of sparks developed in less than 15 ms (Fig. 5C). The average duration was identical with the first peak position of the theoretical Gaussian function (25.52 ± 0.16 ms). The histogram of duration did not show the classical bell shaped curve since the upper limit was chosen arbitrarily (Fig. 5D).

**Figure 6:**
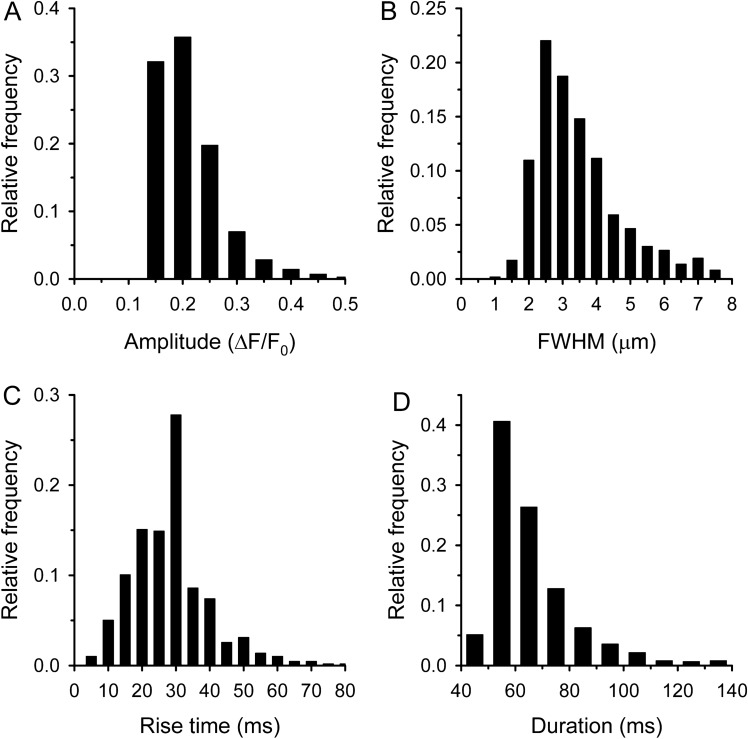
Parameters of calcium embers. Histogram of amplitudes (**A**), FWHMs (**B**), rise times (**C**), and durations (**D**). The number of embers was 1094.

We classified 33% of events as embers with significantly lower frequency than sparks (Table 1). Their average amplitude was identical to that of sparks (0.186 ± 0.002 ΔF/F_0_; p > 0.6). The maximum of ember amplitude was 0.5 ΔF/F_0_ (Fig. 6A). The average FWHM was significantly larger than for sparks (Table 1), likely because calcium had more time to diffuse during embers (Fig. 6B). The average rise time was also significantly longer than for sparks (Table 1) showing that the development of embers is a slower process (Fig. 6C). The average duration was somewhat bigger than the second peak position of the theoretical Gaussian function (56.95 ± 0.56 ms). We found some very long events and more than 5% of the embers were longer than 100 ms (Fig. 6D).

**Figure 7:**
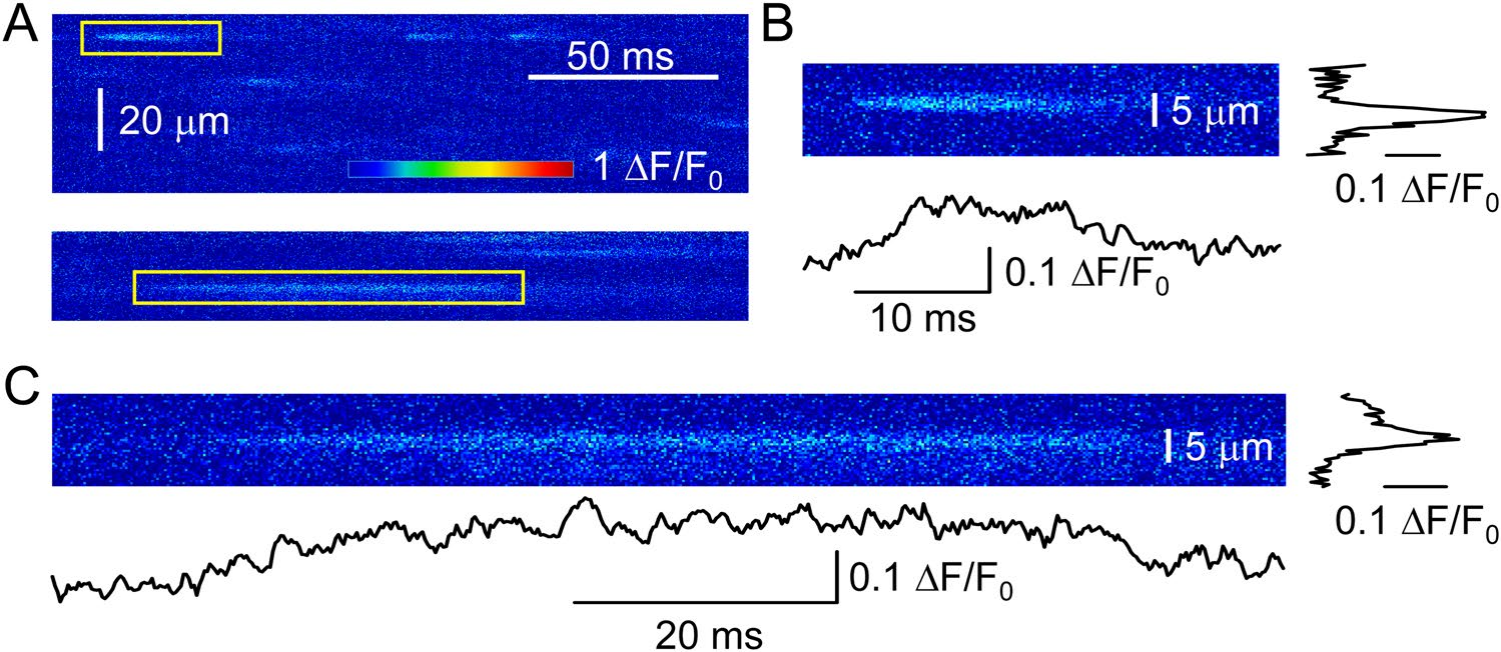
Events on line-scan images at fast scan speed (0.2 ms/line). (**A**) Normalized line-scan images showing sparks (top) and an ember (bottom). Enlarged image of a spark (**B**) and an ember (**C**) with its spatial and time profile. The positions of the events are marked with yellow rectangular in panel A.

High speed confocal scanning was used to study in more details the time profile of CREs (Fig. 7A). This speed (200 µs/line) allowed us to explore the rising and the falling phases of the events. We could confirm our observation that the amplitude of sparks and embers are roughly the same in honey bee muscle. Furthermore if one compares the time profile of a spark (Fig. 7B) to that of an ember (Fig. 7C), it is clear that embers need more time to reach their peak.

## Discussion

In the honey bee leg skeletal muscle, excitation–contraction coupling relies on a calcium entry through plasma membrane voltage-gated calcium channels, which activates ryanodine receptors to release more Ca^2+^ from intracellular stores^5,6^. Whereas macroscopic calcium signalling has been studied in invertebrate species, subcellular calcium release events such as Ca^2+^ sparks have never been described in detail. Our work may help understanding the role and regulation of these events, since interspecific comparisons often lead unravelling cellular phenomena. Data collected in the present study were compared to all available data on CREs recorded in intact freshly isolated skeletal muscle fibres and in intact cell-cultured myotubes, gathered in Tables 2–4. In our comparison, ‘intact’ refers to the integrity of the plasma-membrane, without any attempt at manipulating the intracellular cytoplasmic composition and with physiological extracellular solutions bathing the cells. In honey bee fibres, both sparks (i.e. CREs of short duration) and embers which are CREs of long duration first characterized in adult frog muscle cells^30^, were recorded. Similar short- or long-lasting events have been characterized, for instance in adult mammalian fibres subjected to surface membrane skinning^31^ or in intact foetal mammalians muscle fibres^32^. Embers were seen in intact mouse fibres challenged with depolarization^33^. By comparison, embers are more difficult to detect spontaneously in frog muscle fibres unless pharmacological manipulations were applied^16^.

**Table 2:** Interspecific comparison of calcium sparks’ parameters measured in two dimensional (XY) experiments made in intact cells.

Type of intact cells	Organism	Age (days after birth)	Sarcomere (µm)	Dye	Ext. Sol	[Ca^2+^]_ext_ (mM)	Frame rate (image/s)	Incidence (% cells)	Frequency (events/s/mm^2^)	Amplitude (ΔF/F_0_)	FWHM or EDHM (µm)	Signal mass (µm^3^)	Reference
Adult tibia fibres	Honey bee	1-4	~ 4	Fluo-8-AM	Tyrode	2	1	< 20%	1696 ± 367	0.220 ± 0.001	3.73 ± 0.02 (X) 3.30 ± 0.02 (Y) 3.30 ± 0.02 (Y)	14.52 ± 0.29	This study
Adult FDB fibres	Frog	–	~ 2	Fluo-4-AM	Ringer	1.8	30 and 60	~ 20%^#^	1470 ± 300	0.188 ± 0.001	1.73 ± 0.01 (x) 1.51 ± 0.01 (y)	1.02 ± 0.01	Cserne-Szappanos 2020
Adult FDB fibres	Mouse	> 90	–	Fluo-3-AM	Krebs	2	1.2	< 5%	–	–	–	–	Conklin 1999
Adult fibres*	Mouse	1	–	Fluo-4-AM	Ringer	1.8	1	–	~ 80 (EDL) ~ 55 (D)	~ 0.9 (EDL) ~ 0.9 (D)	1.56 ± 0.04 (EDL) ~ 1.6 (D)	2.17 ± 0.20 (EDL) ~ 2.0 (D)	Chun 2003*
Adult fibres *	Mouse	7	–	Fluo-4-AM	Ringer	1.8	1	–	~ 5 (EDL) ~ 25 (D)	~ 0.9 (EDL) ~ 0.7 (D)	~ 1.25 (EDL) ~ 1.32 (D)	~ 1.2 (EDL) ~ 0.8 (D)	Chun 2003*
Adult fibres *	Mouse	14	–	Fluo-4-AM	Ringer	1.8	1	–	~ 7 (EDL) ~ 5 (D)	~ 1 (EDL) 0.65 ± 0.05 (D)	1.10 ± 0.05 (EDL) ~ 1.3 (D)	0.66 ± 0.12 (EDL) 0.80 ± 0.25 (D)	Chun 2003*
Adult interossei fibres	Mouse	–	–	Fluo-4AM	Ringer	2	0.9	–	15.1 ± 1.2	–	–	–	Teichmann 2008
Foetal intercostal fibres	Mouse	3 days before birth	–	Fluo-3-AM	Krebs	2	1.2	< 20%	–	–	–	–	Conklin 1999
Foetal diaphragm fibres	Mouse	3 days before birth	–	Fluo-4AM	Ringer	1.8	1	~ 20%	~ 80	1.24 ± 0.02	~ 1.67 (D)	3.79 ± 0.22 (D)	Chun 2003*

In this table studies are ordered according to i) the developmental stage (from adult to foetal stages) and ii) the species (Honey bee, Frog and Mouse). All cells were freshly isolated muscle fibres.

*Chun values are from adult P1, P7 and P14 diaphragm (D) and EDL muscle. Foetal diaphragm is from E18, I.e. 3 days before birth.

^#^Personal communication.

Data marked with ~ is approximated from figures. All data depicted at room temperature.

FDB – flexor digitorum brevis; EDL – extensor digitorum longus; D- diaphragm; AM – acetoxymethyl ester; FWHM – full width at half maximum; EDHM – equivalent diameter at half maximum.

A major difference lies in the spatial spreading of CREs from adult bees, which are clearly wider than CREs from adult intact vertebrate muscle fibres (FWHM, Tables 2–4). First, it could be related to the fact that sarcomeres are almost twice as long in bee leg muscle fibres 4.2 ± 0.1 µm^6^, as compared as vertebrate ones (~ 2–2.5 µm). Secondly, insect myofibrils are radially oriented around a central row of nuclei, (an arrangement which is not seen in adult vertebrate muscle fibres), giving a so-called ‘tubular’ morphology to the fibres. Accordingly, insect tubular muscle fibres have relatively straight t-tubules arranged in radial symmetry from the surface membrane to central nuclei in order to convey action potentials throughout the fibre volume^34^. This spatial configuration may lead to less restricted diffusion along long portions of straight T-tubules. The average ratio of FWHMs (X/Y) honey bee CREs was greater than 1 (1.23 ± 0.01), which indicates that ellipsoidal sparks diffuse slightly more freely in the X direction (along the long axis of the fibre) than in the Y direction (along T-tubules). Elongated sparks were detected in frog skeletal muscle as well^22,35^ and CREs non-circularity has been previously attributed to a ‘channelled diffusion’ due to tubes of mitochondria^17^, but this hypothesis remains to be explored in bee fibres. Thirdly, an interesting feature is that bee T-tubules are running at the junction of A and I bands, like in mammalian skeletal muscle fibres, so that two T-tubules run per sarcomere^5^, increasing the chance to recruit release sites for large CREs. Fourthly, electron microscopy and rotary shadowing techniques showed that RyR disposition in invertebrate muscle differ from their disposition in vertebrates^36^, an arrangement that may favour wider CREs in honey bees. Finally, ultrastructural differences were also identified in the invertebrate couplons, i.e. the functional arrangement of RyRs and voltage-gated calcium channels from the plasma membrane (DHPRs, for dihydropyridine receptors), and previous studies underlined that this architecture was reminiscent of the cardiac striated muscle^37^. Accordingly, other ultrastructural similarities can be pointed out in between bee skeletal and vertebrate cardiac muscles, such as the presence of dyads in both tissues instead of the triads observed in vertebrate skeletal muscles^5^. Besides structural and ultrastructural differences, that clearly settle the conditions for broad CREs in insect muscle fibres, genomic differences could favour it as well. Homology data on RyR sequences suggest that invertebrates and vertebrates show important genomic differences. First, insects have only one *ryr* gene whereas amphibians have two (*ryr* α also named ryr 1, and *ryr* β also named *ryr* 3) and mammalians have three^38,39^. Two isoforms are actually expressed in vertebrate skeletal muscles (*ryr* 1 and *ryr* 3 in mammalians and amphibians), since the *ryr* 2 gene is expressed in cardiac mammalian muscle only. After the ‘honey bee genome sequencing consortium’ made available the honey bee (*Apis mellifera*) genome sequence^40^, the predicted amino acid sequence for the *Apis mellifera* RyR protein (*Am*RyR) has been obtained from an annotated genomic sequence NC_037639.1 (Gene 408680 on chromosome LG2) using traditional gene prediction methods (GNOMON, supported by EST evidence). Like in other species, predicted *Am*RyR protein isoforms (X1 to X9) contain ~ 5000 amino acids. According to BLAST tools (NCBI database), the homology degree of the honey bee *Am*RyR with vertebrate RyRs is around 45%. Such a difference may well settle the basis for strong differences into the protein functions and regulations leading to morphological differences in bee CREs as compared to other species. In zebrafish skeletal muscle cells, two RyR isoforms have been shown to be essential to achieve normal sparks production^41^. The second frog RyR isoform may also be related to high spark production in this species^42^. In foetal mammalian muscles, the two isoforms of RyR (1 and 3) are expressed and it was suggested that they both contribute to generate sparks^43^. Conversely, at the mature adult stage, most intact mammalian skeletal fibres (which no longer express RyR3) require specific conditions to fire sparks and at a relatively low frequency. Our data show that a single *ryr* gene allows the production of a high CREs frequency with wide spatial spread in intact insect fibres.

Functional differences could also lead to the specific shape properties of bees CREs. In the honeybee muscle, as in other invertebrates, excitation–contraction coupling relies on i) Ca^2+^ entry through the plasmalemmal membrane via voltage-gated channels and ii) on a calcium induced calcium release (CICR) mechanism^5,44^. CICR is thus a crucial mechanism in the skeletal muscle from bees and other insects (which are devoid of TTX-sensitive voltage-gated channels) where voltage-gated calcium currents activate quickly to achieve a rapid action potential overshoot^6,45,46^. The role of CICR in excitation–contraction coupling implies that honey bee RyRs must be highly sensitive to Ca^2+^, a feature that favours broad CREs. CICR has been shown in frog muscle fibres as well (with a crucial role attributed to the second RyR isoform) but it plays a null or minor role in rat fibres^47^. Despite a demonstrated CICR in frog, CREs from frog fibres are narrower than CREs from mammalian fibres, both in intact cells (Table 3) or in permeabilized fibres^48^. In the two vertebrate species, CREs width is narrower than in bees, which is clearly in agreement with a more important role for CICR in insects. In half of adult bee leg muscle fibres, a low-voltage-activated (LVA) current has been identified in addition to the nifedipine-sensitive high-voltage activated (HVA) current^5,49^. Both HVA- and LVA- calcium currents exists in mammalian fibres as well, but the density of the later gradually decreases postnatally to eventually disappear after 2–3 weeks of age^50^. Whereas DHPRs responsible for the HVA- (L-type) current are involved in triggering and/or repressing sparks in immature and adult mammalian muscle fibres^27,32^, pharmacological proofs indicate that the LVA- (T-type) calcium channel doesn’t modulate sparks frequency^32^. A role for the LVA- current in the genesis or regulation of honey bee CREs thus remains to be studied.

**Table 3:** Interspecific comparison of calcium sparks’ and depolarization-evoked transients’ parameters measured in line-scan (XT) experiments performed in intact cells.

Type of intact cells	Organism	Age (days)	Sarcomere (µm)	Dye	Ext. Sol	[Ca^2+^]_ext_ (mM)	Speed of recording (line/ms)	Frequency (events/ /s/mm)	Sparks amplitude (ΔF/F_0_)	FWHM (µm)	FDHM or FTHM (ms)	Rise time (ms)	Signal mass (µm^3^)	Depolarization-induced macroscopic Ca^2+^transient (ΔF/F_0_)	Reference
Adult tibia fibres	Honey bee	1-4	~ 4	Fluo-8-AM	Tyrode	2	1	174.0 ± 14.4	0.185 ± 0.001	2.65 ± 0.02	25.52 ± 0.16	10.45 ± 0.11	6.00 ± 0.20	1.5 ± 0.16	This study
Adult iliofibularis and semitendinosus fibres	Frog	–	~ 3 ǂ	Fluo-3 (injected)	Ringer	1.8	2.048	23 ± 9	0.92 ± 0.02	0.95 ± 0.04	5.66 ± 0.23	0–100% rise time 3.49 ± 0.15	1.28 ± 0.17	18.6 ± 0.9	Hollingworth 2001
Adult FDB fibres	Mouse	> 90	–	Fluo-3-AM	Krebs	2	2.05	< 20	0.9 ± 0.3	2.0 ± 0.5	40.3 ± 35	16.4 ± 14	–	–	Conklin 1999
Adult fibres	Mouse	1	–	Fluo-4-AM	Ringer	1.8	2	–	0.7 ± 0.1 (EDL) 0.8 ± 0.1 (D)	1.5 ± 0.1 (EDL) 0.7 ± 0.2 (D)	13.2 ± 0.7 (EDL) 10.5 ± 1.1 (D)	8.9 ± 0.7 (EDL) 8.0 ± 1.2 (D)	–	–	Chun 2003*
Adult fibres	Mouse	7	–	Fluo-4-AM	Ringer	1.8	2	–	0.6 ± 0.1 (EDL) 0.7 ± 0.1 (D)	1.7 ± 0.1 (EDL) 1.6 ± 0.2 (D)	12.6 + 0.9 (EDL) 11.3 ± 1.1 (D)	7.3 ± 0.7 (EDL) 6.1 ± 0.6 (D)	–	–	Chun 2003*
Adult fibres	Mouse	14	–	Fluo-4-AM	Ringer	1.8	2	–	1.5 ± 0.5 (EDL) 0.5 ± 0.1 (D)	1.6 ± 0.2 (EDL) 1.5 ± 0.1 (D)	13.5 + 1.6 (EDL) 11.2 ± 0.9 (D)	9.3 ± 1.4 (EDL) 7.6 ± 1.3 (D)	–	–	Chun 2003*
Foetal fibres	Mouse	3 days before birth	–	Fluo-3-AM	Krebs	2	2.05	< 20	1.6 ± 0.6	3.5 ± 1.1	84.5 ± 46	42.8 ± 24	–	–	Conklin 1999
Foetal fibres	Mouse	3 days before birth	–	Fluo-4-AM	Ringer	1.8	2	–	0.9 ± 0.1	1.9 ± 0.1	13.9 ± 0.4	Rise(10–90%) 7.6 ± 0.3	–	–	Chun 2003*
Larval tail fibres	Zebrafish	3 days postfertilization	~ 1.5	Fluo-4-AM	Ringer with 0.3 mM Caffeine	2	–	~ 44	~ 0.8	~ 1.3	~ 5.2	~ 4	~ 3	–	Perni 2015
Primary myotubes culture	Mouse	–	–	Fluo-3-AM	TEA-MeSO_3_	2	2	–	0.94 ± 0.05	2.1 ± 0.3	11.8 ± 1.6	–	–	–	Shirokova 1998
Primary myotubes culture	Rat	~ 5 days in vitro	–	Fluo-4-AM	Tyrode	1.8	1.54	3.2 ± 1.9	0.49 ± 0.03	1.67 ± 0.16	39.7 ± 4.3	15.4 ± 2.2	–	0.62 ± 0.05	Fodor 2008
C2C12 cell line myotubes	Mouse	3–6 days in vitro	–	Fluo-3-AM	Ringer	10	0.02–007	0.3	~ 300 nM	–	–	–	–	–	Gyorke 1996
C2C12 cell line myotubes	Mouse	> 5 days in vitro	–	Fluo-4-AM	Tyrode	2	0.8	16	0.49 ± 0.01	3.8 ± 0.08	92.8 ± 8.7	55.9 ± 4.2	–	~ 2.5	Altomare 2013^$^

In this table studies are ordered according to i) the developmental stage (from adult to foetal/larval stages) and ii) the species (Honey bee, Frog, Mouse, Rat, Zebrafish). All preparations were freshly isolated muscle fibres except the last four where the cells were in cell culture.

*Chun values are from P1, P7, and P14 EDL muscle and from foetal (3 days before birth) diaphragm, rise time was calculated between 10–90% of the peak amplitude.

ǂsarcomere stretched to ~ 3 µm.

^$^parameters of CREs originate from sparks and embers together.

Data marked with ~ is approximated from figures. All data depicted at room temperature except Perni et al. 2015 (18 °C). Hollingworth et al. 2001 cooled the fibres to 17–20 °C.

All studies were from intact cells bathed in a physiological solution (Ringer, Krebs or Tyrode), except for Perni et al. 2015 (caffeine added) and Shirokova et al. 1998 (150 TEA-MeSO_3_) ^57^.

FDB – flexor digitorum brevis; EDL – extensor digitorum longus; D- diaphragm; AM – acetoxymethyl ester; FWHM – full width at half maximum; FDHM – full duration at half maximum; FTHM – full time at half maximum.

Amplitude of honey bee sparks measured in *XYT* experiments appeared smaller than in almost all other intact preparations (Table 2, 3). This parameter is however highly subjected to the experimental set-up performances, imaging analysis and thresholding strategies, as illustrated in *XT* experiments from frog, where amplitude can vary by a factor 5 (Table 3). Namely, using the traditional threshold-based sparks detection method, the former study of sparks in intact frog fibres described an average amplitude of ~ 1 ΔF/F_0_^23^, whereas a recent publication using the wavelet detection method described an average amplitude of ~ 0.2 ΔF/F_0_^22^. The difference may be in part attributed to the more efficient detection of CREs of low amplitude with the wavelet method, but other factors may be involved because the maximal amplitudes detected in the later publication reached ~ 0.85 ΔF/F_0_^22^. Interestingly, in intact muscle fibres from a mouse model of myopathy (*mdx*), a similar analysis strategy (‘à trous implementation of the two-dimensional undecimated discrete wavelet transform’) led to the detection of CREs up to 1.3 ΔF/F_0_, with a half cumulative frequency value (CF_50_) of 0.53^51^. The apparently low amplitude of honey bee sparks as compared to canonical sparks may thus be more related to species specificities and/or experimental conditions than to the detection method we used. Another striking difference between bees and vertebrates is that in bees, embers had almost the same amplitude as sparks, whereas embers are usually smaller than sparks in mammals, as shown in non-intact preparations^16,31^. This finding implies the idea that in bee muscle during a CRE, the same number of RyRs open and only the duration of their opening vary stochastically. The amplitude of sparks from honey bee is ~ eightfold smaller than the amplitude of voltage-gated global Ca^2+^ transients (Table 3), a result consistent with the idea that sparks are the unitary building blocks of macroscopic calcium release. This ratio is in the same range in intact frog fibres (in *Rana pipiens* fibres, sparks are ~ 20 smaller than voltage-activated macroscopic transients^23^). In intact mouse primary myotubes and in the C2C12 mouse cell line, sparks are 1.4 and ~ fivefold smaller than macroscopic transients, respectively^52,53^. To the best of our knowledge, no study relates both sparks amplitude and macroscopic transients in intact non voltage-clamped mouse fibres. A ratio of ~ 6 can however be calculated from macroscopic Ca^2+^ transients recorded under similar conditions as those depicted in Table 3 for fibres of the mouse FDB muscle^54^.

Honey bee sparks had similar kinetics properties as other species. Most features of honey bee embers were reminiscent to other species ones as well. Previous studies classified the embers using their durations (Table 4). Although there was no clear border between the duration of sparks and embers in bee muscle (see Fig. 4D), the distribution of the probability histogram of bee CREs duration was nevertheless successfully fitted by a bimodal Gaussian function, according to a previously described methodology^55^ and the value 40 ms was retained as the threshold for ember detection. Importantly, the amplitude could not be used as the second separator parameter since CREs in bee fibres are more homogeneous in amplitude than in mammalian muscles where the amplitude of embers is much smaller than that of the sparks.

**Table 4:** Interspecific comparison of calcium embers’ parameters measured in line-scan (XT) experiments performed in intact cells.

Type of intact cells	Organism	Age (days)	Sarcomere (µm)	Dye	Ext. Sol	[Ca^2+^]_ext_ (mM)	Speed of recording (line/ms)	Threshold for duration of embers (ms)	Frequency (events/ /s/mm)	Amplitude (ΔF/F_0_)	FWHM (µm)	Duration (ms)	Rise time (ms)	Signal mass (µm^n3^)	Reference
Adult tibia fibres	Honey bee	1–4	~ 4	Fluo-8 AM	Tyrode	2	1	40	63.1 ± 6.6	0.186 ± 0.002	3.18 ± 0.04	56.95 ± 0.58	27.02 ± 0.41	10.29 ± 0.41	This study
Adult FDB fibres	Mouse	1–14	–	Fluo-4 AM	Ringer	1.8	2	30	~ 4% of CREs ǂ	–	–	–	–	–	Chun 2003*
Primary myotubes culture	Rat	~ 5 days in vitro	–	Fluo-4 AM	Tyrode	1.8	1.54	60	2.4 ± 1.4	0.27 ± 0.05	-	229 ± 33	–	–	Fodor 2008
C2C12 cell line myotubes	Mouse	> 5 days in vitro	–	Fluo-4 AM	Tyrode	2	0.8	50	53.4% of CREs ǂ	–	–	–	–	–	Altomare 2013

In this table studies are ordered according to i) the developmental stage and ii) the species (Honey bee, Frog, Mouse, Rat). Preparations were freshly isolated muscle fibres or cell culture.

*Chun values are from EDL and diaphragm muscles. Rise time was calculated between 10–90% of the peak amplitude.

ǂThe embers frequency was given as a percentage of total CRE in these studies.

Data marked with ~ is approximated from figures. All data depicted at room temperature.

FDB – flexor digitorum brevis; AM – acetoxymethyl ester; FWHM – full width at half maximum.

To our knowledge, few studies depict incidence of sparks in intact skeletal muscle (i.e. the percentage of fibres where such events can be detected). CREs were seen in < 20% of adult honey bee intact fibres. By comparison, the incidence of spontaneous CREs was less than 5% of intact adult mouse fibres whereas it was more elevated in embryonic fibres^43^. A comparison of sparks frequency collected in *XT* line-scan experiments in intact cells shows that in honey bee, frequency is four fold to 600 fold higher than in any other mature or immature cells from frog, mouse, rat or zebrafish (Table 3, *XT*). In *XYT* images series from adult bees’ fibres, the frequency is one or two orders of magnitude higher as compared to intact cells from adult or immature mammalian cells (20–300 fold higher, Table 2). For instance, in mouse cells the frequency of CREs in *XYT* has been described to be between 5 and 80 Hz/mm^2^ only (Table 2, *XYT*). Interestingly, the *XYT* frequency observed in bees is almost identical to what was reported in intact adult frog muscle recently^22^. Again, these differences in CREs frequency among species may thus be a consequence of the relative incidence that CICR has in excitation–contraction coupling or to ultrastructural differences discussed before. It is important to point out that frequency similarities between bees and frogs are not only related to the analysis strategy, because a similar procedure used in mouse fibres led to a 100 fold lower frequency^51^. Finally, the proportion of embers as compared to the total number of CREs in *XT* experiments shows that honey bee embers are much more frequent than in adult or foetal mouse cells (33% in honey bee vs 4% in mouse) and either higher or identical in myotubes from rat and mouse respectively (Table 4).

In honey bee fibres, CREs appear most of the time in a stochastic manner and rarely at a given triad. However, a repetitive mode of activation was observed in specific hot spots. This second mode, previously termed *rep*-mode, has been identified when voltage-clamped vertebrate fibres (cut fibres mounted in a Vaseline gap chamber) were stimulated by a low-amplitude depolarization^56^. This activity mode can also happen in chronically depolarized frog fibre right after a brief repolarization protocol. Such an activity was seen in intact adult mouse fibres as well^43^ and in embryonic and postnatal mouse fibres, where 29% of all events location had reoccurring Ca^2+^ release events^32^. A pharmacological manipulation with caffeine is also increasing the appearance of the repetitive CREs in intact frog fibres^22^. However, whereas CREs in rep-mode were short in duration in frog fibres, bee hot spots led to CREs with various kinetics ranging from sparks to burst and embers.

In addition to provide the first interspecific comparison of sparks between vertebrates and invertebrates, the present study sets the basis for a future comparison of sparks in other invertebrates, where these subcellular localized Ca^2+^ signals have not been described yet. We have overcome the difficulty of isolating single fibres from insects (firmly attached to the insect exoskeleton through tonofilaments), and this methodological advancement will allow an in-depth characterization of sparks in insects in general. Our methods could in particular be transposed to common insect models used either in fundamental research (fruit flies, cockroaches, grasshoppers) or models of interest in agronomy (aphids, moths, bugs, etc.). This study would be of particular interest to characterize the interspecific difference in susceptibility to new insecticides targeting RyRs, the phthalic acid diamides and anthranilic diamides. Diamides are highly efficient to control a variety of pests. They are widely used to kill a number of insect species, including lepidopterans (moths), hemipterans (aphids), dipterans (flies) and so on. Their major mode of action is to interfere with normal function of ryanodine receptors. They have been designed to ensure a favourable vertebrate selectivity ratio, that is to say: being more toxic to invertebrates than to vertebrates. However, insects exposed to these compounds, whether they are considered either as pests or beneficial insects, show strong symptoms of intoxication (from sublethal effects to mortality). For instance, we have shown that sublethal doses of chlorantraniliprole were highly deleterious to honey bees that encounter long lasting locomotor deficits lasting for several days, leading to high susceptibility to other threats^9^. Higher doses kill the bees quickly. Insect ryanodine receptors are quite divergent from those of vertebrates (~ 45% a.a. identity), but RyRs from insect species from all orders show a strong molecular proximity (~ 80%). The honeybee RyR (XP_006569098.1) shows 83, 80, 79 and 78% amino acid identity with RyRs from the beetle *Tribolium castaneum* (NP_001308588.1), the moth *Plutella xylostella* (XP_037970554.1), the aphid *Myzus Persicae* (XP_022160123.1) and the fly *Drosophila melanogaster* (NP_476991.1), respectively. Although fruit flies *D. melanogaster* are considered as models, the intracellular homeostasis in insect tubular muscle fibres has so far not been studied thoroughly owing to the difficulty of isolating muscle fibres from the cuticle, which is a prerequisite for calcium imaging. The honey bee fibres provide a suitable model in this context and the dissociation procedure we developed here to study sparks will be helpful in many instances.

## Conclusion

In addition to the characterization of localized Ca^2+^-release events of the honey bee, a detailed comparison of their major specificities as compared with their vertebrate counterparts has been carried out. The successful isolation of insect muscle fibres and the thorough characterization of subcellular Ca^2+^ signalling in the honey bee muscle paves the way for further in vitro studies, which are clearly needed to elucidate the origins of the established differential toxicity of the RyR-targeting diamides insecticides between vertebrates and invertebrates. Furthermore, while a so-called ‘vertebrate selectivity toxicological ratio’ already exists for diamides, no protective selectivity ratio has been established yet for useful insects. Pest insects and non-pest insects share a high degree of homology in their RyR and a comparative study of Ca^2+^ sparks among insects may help to better characterize the toxicity of diamides, which constitute a threat to useful insects such as pollinators.

## Supplementary Information

Supplementary file 1.

Supplementary Video 1.

## Data Availability

The datasets generated and analysed during the current study are available on reasonable request.
