# Supplementary figures and images for: Elementary calcium release events in the skeletal muscle cells of the honey bee Apis mellifera
Source: Sci Rep. 2021 Aug 18;11:16731. doi: 10.1038/s41598-021-96028-w (PMC8373864; doi:10.1038/s41598-021-96028-w)

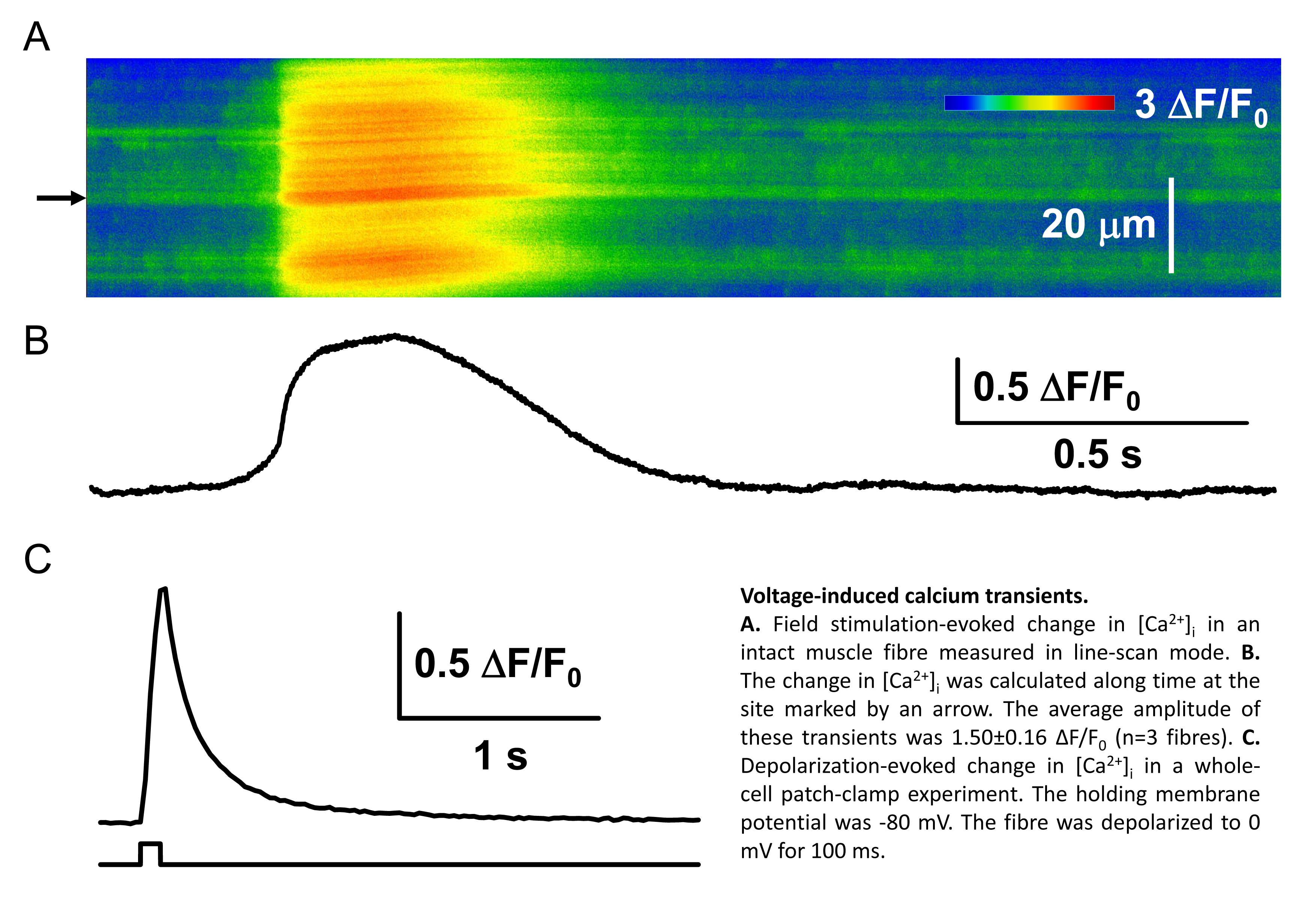

Supplement: Supplementary file 1 [file 41598_2021_96028_MOESM1_ESM.tif]
